# Morin Hydrate Sensitizes Hepatoma Cells and Xenograft Tumor towards Cisplatin by Downregulating PARP-1-HMGB1 Mediated Autophagy

**DOI:** 10.3390/ijms21218253

**Published:** 2020-11-04

**Authors:** Mahendra Pal Singh, Tejinder Pal Khaket, Vivek K. Bajpai, Saleh Alfarraj, Se-Gie Kim, Lei Chen, Yun Suk Huh, Young-Kyu Han, Sun Chul Kang

**Affiliations:** 1 Department of Biotechnology, Daegu University, Gyeongsan, Gyeongbuk 38453, Korea; mahendra.rathore9@gmail.com; 2 Department of Immunology, Mayo Clinic, Rochester, MN 55905, USA; 3 Department of Radiation Oncology, The Ohio State University, Columbus, OH 43210, USA; tejkhaket@gmail.com; 4 Department of Energy and Materials Engineering, Dongguk University-Seoul, 30 Pildong-ro 1-gil, Seoul 04620, Korea; vbiotech04@gmail.com; 5 Zoology Department, College of Science, King Saud University, Riyadh 11451, Saudi Arabia; salfarraj@hotmail.com; 6 Department of Pharmaceutical Engineering, Daegu Catholic University, Gyeongsan, Gyeongbuk 38430, Korea; sgkim7@cu.ac.kr; 7 College of Food Science, Fujian Agriculture and Forestry University, Fuzhou 350002, China; 8 Department of Biological Engineering, Biohybrid Systems Research Center (BSRC), Inha University, 100 Inha-ro, Nam-gu, Incheon 22212, Korea; yunsuk.huh@inha.ac.kr

**Keywords:** apoptosis, autophagy, cisplatin, HMGB1, morin hydrate

## Abstract

The cross-talk between apoptosis and autophagy influences anticancer drug sensitivity and cellular death in various cancer cell lines. However, the fundamental mechanisms behind this phenomenon are still unidentified. We demonstrated anti-cancerous role of cisplatin (CP) and morin hydrate (Mh) as an individual and/or in combination (CP-Mh) in hepatoma cells and tumor model. Exposure of CP resulted in the production of intracellular reactive oxygen species (ROS)-mediated cellular vacuolization, expansion of mitochondria membrane and activation of endoplasmic reticulum (ER)-stress. Consequently, Cyt *c* translocation led to the increase of Bax/Bcl-2 ratio, which simultaneously triggered caspase-mediated cellular apoptosis. In addition, CP-induced PARP-1 activation led to ADP-ribosylation of HMGB1, which consequently developed autophagy as evident by the LC3I/II ratio. Chemically-induced inhibition of autophagy marked by increased cell death signified a protective role of autophagy against CP treatment. CP-Mh abrogates the PARP-1 expression and significantly reduced HMGB1-cytoplasmic translocation with subsequent inhibition of the HMGB1-Beclin1 complex formation. In the absence of PARP-1, a reduced HMGB1 mediated autophagy was observed followed by induced caspase-dependent apoptosis. To confirm the role of PARP-1-HMGB1 signaling in autophagy, we used the PARP-1 inhibitor, 4-amino-1,8-naphthalimide (ANI), HMGB1 inhibitor, ethyl pyruvate (EP), autophagy inhibitors, 3-methyl adenine (3-MA) and bafilomycin (baf) and small interfering RNAs (siRNA) to target Atg5 in combination of CP and Mh. Exposure to these inhibitors enhanced the sensitivity of HepG2 cells to CP. Collectively, our findings indicate that CP-Mh in combination served as a prominent regulator of autophagy and significant inducer of apoptosis that maintains a homeostatic balance towards HepG2 cells and the subcutaneous tumor model.

## 1. Introduction

Hepatocellular carcinoma (HCC) accounts for the third leading cause of cancer mortality worldwide. In Korea, 12% of all malignancies account for HCC and it is one of the leading causes of cancer-related deaths [1]. To date, there is no definitive curative drug for the treatment of HCC. Chemotherapy is often used to control tumor progression when it reaches an unresectable condition [2], however, there is an increasing development of drug-resistance and poor responders to the available conventional drugs. Hence, the development of a novel chemotherapeutic drug or a combinational therapy that improves the efficacy and sensitivity towards cancer with the currently available drugs is an area of intense research.

Cisplatin (CP) is a commonly used single chemotherapeutic agent with modest anti-tumor potential and safety in patients with HCC [3]. The anti-tumor effect of CP is attributed to the formation of adducts between inter and intra-strands of DNA. The cytotoxic effect of CP is likely to the results of the inhibition of DNA replication and induction of apoptosis. Nevertheless, its efficacy is incessantly limited due to the high incidence of chemo-resistance [4]. Therefore, rather than developing a new drug, it is imperative to develop newer methods to conquer the drug-resistance. We believe that combination therapy might be effective in preventing drug-resistance by altering the expression of different proteins.

Recent studies have investigated the role of multiple signaling pathways responsible for CP-resistance in a variety of cancers, of which PARP-1-HMGB1 pathway-mediated autophagy activation plays a key role [5,6]. PARP-1 is an intra-nuclear enzyme, which leads to the conversion of β-nicotinamide adenine dinucleotide (NAD^+^) to the polymer of poly (ADP ribose) (PAR) to regulate nuclear homeostasis. Chemical induced DNA damage in cells activates PARP-1, which depletes NAD^+^ and ATP, subsequently leading to irreversible energy scarcity and cell death [7]. The physio-pathological role of PARP-1 hyper-activation through DNA damage is well exemplified by using PARP-1 inhibitors in the experimental model of diabetes, inflammation and cancer [8]. The role of poly-ADP-ribosylation in cell death shows that PARP-1 activation occurs during apoptosis. Despite the pathogenic relevance, PARP-1 leads to poly-ADP-ribosylation of HMGB1, which in turn contributes to chemotherapy resistance via upregulation of autophagy through three different mechanisms; (1) nuclear HMGB1 activates autophagy by HSPB1 (heat shock 27 kDa protein 1) regulation, (2) cytoplasmic HMGB1 binds Beclin1 to initiate autophagosome formation and (3) extracellular HMGB1 leads to advanced glycation end product-specific receptor (AGER)-mediated activation of class III phosphatidylinositol 3-kinase to induce autophagy [9]. Since alterations in the expression of HMGB1 and its sub-cellular translocation are associated with the regulation of tumor development and cancer treatment, we propose that HMGB1-mediated autophagy activation is a possible target for chemotherapy [10]. In our previous study, we have reported the regulatory role of morin hydrate (Mh) in CP-induced autophagy activation induced by PARP-1 activation in CP-resistance HepG2^DR^ multi-drug resistant cell line. According to these findings, the cisplatin/morin hydrate combination was effective in the reversal of the HepG2^DR^ cell resistance via suppression of PARP1-mediated autophagy by regulating the HMGB1 and microtubule-associated protein 1A/1B light chain 3B (LC3) I/II [11]. In continuation of our previous report, present study validates the adjuvant effect of Mh in combination with CP to regulate the PARP-1-HMGB1 pathway of autophagy and enhances apoptosis in various HCC cell lines and in xenograft mice model of HCC.

Morin hydrate (Mh) or (3,5,7,2′,4-pentahydroxyflavone) is a bioflavonoid, naturally isolated from the *Moraceae* family members, mostly found in different small plants, fruits and wine [12]. In recent studies, Mh has exhibited several pharmacological properties, including anti-oxidant, anti-inflammatory, apoptosis, anti-proliferative and chemo-sensitivity in multiple cancer cell lines. Mh supplementation to cancer xenograft model of rodents significantly attenuates tumor development by reducing oxidative damages generally induced by free radicals [13,14]. The chemical structure of Mh can be distinguished from other bioflavonoids by the presence of two aromatic rings interconnected by a *c*-pyrone ring with polar hydroxyl groups at various positions. These hydroxyl groups are thought to be harboring free radical scavenging properties shared by Mh and other bioflavonoids [15]. On the other hand, Mh also possesses a characteristic feature of minimal toxicity even at higher dose usage [16]. Based on the demonstrated anti-tumor activities, different mechanisms of action and toxicity profiles, we intended to study combinational therapeutic effect of CP and Mh in HepG2 cells and anticipated a synergistic interaction of CP-Mh combination.

## 2. Results

### 2.1. MH Promotes Chemo-Sensitivity of HepG2 Cells to CP

**Figure 1:**
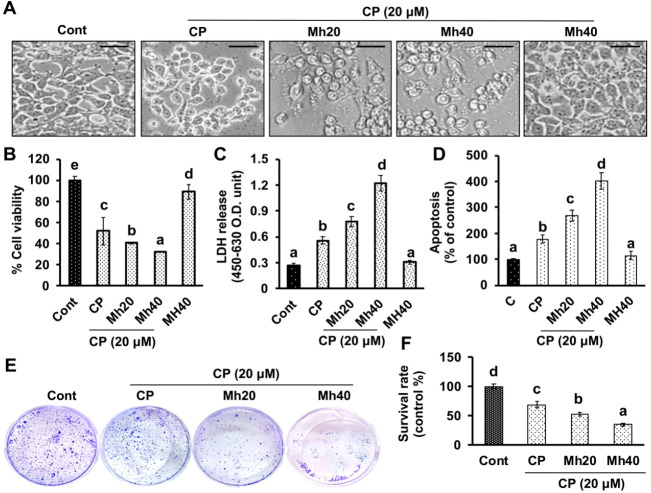
Effect of cisplatin (CP), Mh and CP-Mh on HepG2 cell viability. (**A**) The microscopic images of HepG2 cells morphology after corresponding drug treatment (scale bar 0.1 mm). (**B**) HepG2 cells viability measurement by 3-(4,5-dimethylthiazol-2-yl)-2,5-diphenyltetrazolium bromide (MTT) assay. (**C**) Total LDH release assay (**D**) Apoptosis assay. (**E**) Cell proliferation was evaluated by colony formation assay. (**F**) Colony survival rate. The data are represented as the means standard deviation (±SD, n = 3). The values of different letters (a–d) differ significantly from each other (*p* < 0.05).

To assess the cytotoxicity of individual CP (20 μM), MH (40 μM) and combined CP-Mh on HepG2 cells, we first made the morphological measurement followed by MTT assay assessment. As shown in Figure 1A, CP-Mh treatment resulted in a concentration-dependent morphological changes in HepG2 cells such as cell blebbing, shrinkage, distorted shape and formation of the cytoplasmic vacuoles, which are typical characteristics of apoptosis. CP-Mh combination treatment also significantly increased the rate of cell death in comparison to CP and Mh alone (Figure 1B). To further confirm the cytotoxic effect of CP-Mh, we performed the lactate dehydrogenase (LDH) assay. Our results revealed that exposure to CP-Mh induced significant LDH release in comparison to CP or MH alone treated HepG2 cells (Figure 1C). In addition, the percent cell viability of Huh7, Hep3B and SK-Hep1 cells was also reduced significantly in a concentration-dependent manner after CP-Mh treatment in comparison to untreated and CP-treated cells (Figure S1A–C). To assess the apoptotic cell death, the apoptosis assay was done using the ApoStrand™ ELISA apoptosis detection kit. As presented in Figure 1D, significantly increased DNA denaturation was observed by monoclonal antibodies when cells were exposed to CP-Mh in comparison to CP, MH and untreated HepG2 cells. Furthermore, the clonogenic assay of HepG2 cells evaluated the anti-proliferative role of CP-Mh. As shown in Figure 1E,F, the number of colonies after CP-Mh-treatment were significantly reduced when compared with CP and untreated control HepG2 cells. Obtained data are consistent with our published results and support to validate our previous findings [11].

### 2.2. Mh Suppresses Oxidative-Stress and Induces Mitochondrial Stress in CP-Treated HepG2 Cells

**Figure 2:**
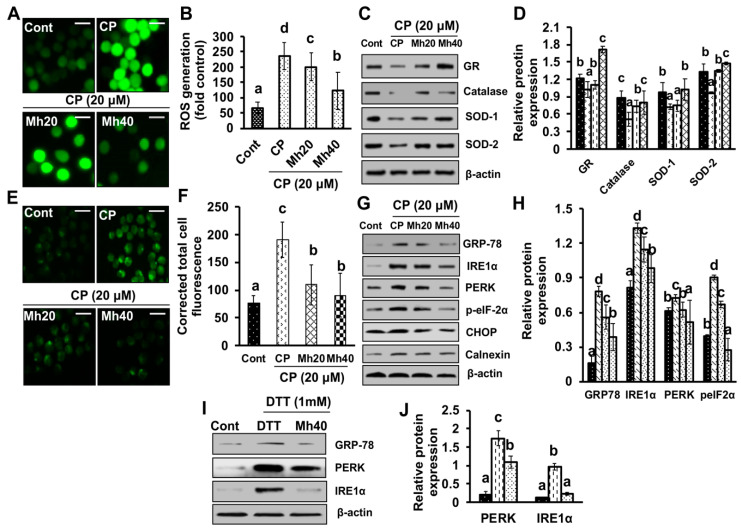
Effect of CP and CP-Mh on cellular oxidative stress. (**A**) Cellular reactive oxygen species (ROS) levels in HepG2 cells after corresponding drug treatment were visualized by fluorescence microscopy (scale bar 0.1 mm). (**B**) Corresponding ROS fluorescence intensity was measured manually by Image J software. (**C**) Western blot analysis of oxidative stress-related markers was done using specific antibodies for GR, catalase, SOD-1 and SOD-2, with β-actin used as an internal loading control. (**D**) Relative expression of GR, catalase, SOD-1 and SOD-2, was analyzed by densitometry analysis by ImageJ software. (**E**) HepG2 cells were stained with Fura-2AM after relevant drug treatment and assayed under a fluorescence microscope (magnification 400×, scale 0.1 mm). (**F**) Intracellular Ca^++^ accumulation was quantified by ImageJ software. (**G**) ER stress markers, including GRP78, IRE1-α, PERK, p-eIF2-α, CHOP and Calnexin, were analyzed by Western blotting. Results were normalized by β-actin in respective of internal controls. (**H**) Relative protein expression was analyzed by densitometry analysis using ImageJ software. (**I**) ER stress markers including GRP78, PERK and IRE1-α, after corresponding drug and DTT treatment were analyzed by Western blotting. β-actin used as an internal loading control. (**J**) Relative protein expression was evaluated by densitometry analysis using ImageJ software. The data are represented as the means standard deviation (±SD, *n* = 3). The values of different letters (a–d) differ significantly from each other (*p* < 0.05).

Oxidative stress plays a critical role in ER stress-induced cell death. To assess whether CP-Mh causes oxidative stress in HepG2 cells, we measured alterations in the intracellular level of ROS in response to CP-Mh following H_2_DCFDA staining. As shown in Figure 2A,B, significantly elevated level of ROS was observed after CP treatment at a given concentration, which was reduced in CP-Mh-treated HepG2 cells with respect to control. To overcome the redox environment, cells maintain complex systems by overlapping the antioxidant enzymes such as superoxide dismutases, glutathione reductase and catalase. In the present study, we demonstrated the effect of CP-Mh on the antioxidant system of HepG2 cells. As shown in Figure 2C,D, significantly reduced expression levels of catalase, glutathione reductase (GR), SOD-1 and SOD-2 were observed in HepG2 cells after CP treatment, which were markedly increased in CP-Mh-treated cells in a concentration-dependent manner when compared with control. Current findings are consistent with our published data which have been obtained through HepG2^DR^ drug resistance cell lines [11].

In order to determine the effect of CP and CP-Mh on intracellular ROS generation and its participation in activation of ER stress signaling, we used the Fura-2AM stain to measure the cytoplasmic Ca^++^ release after exposure to CP and CP-Mh. As shown in Figure 2E,F, enhanced green fluorescence intensity indicates cytoplasmic Ca^++^ release in CP-treated HepG2 cells, which was reduced significantly in CP-Mh-treated cells when compared with CP-treated and control cells. Further, we assessed the effect of CP and CP-Mh on the expression of ER stress-inducing markers such as PERK, IRE1-α, p-eIF2-α, CHOP and calnexin. As shown in Figure 2G,H, the expression levels of the above-stated markers were significantly increased after 24 h of CP treatment, however, markedly reduced in CP-Mh-treated HepG2 cells in a concentration-dependent manner with respect to control. Moreover, to determine whether Mh suppresses ER stress, we measured the expression of GRP-78, PERK and IRE1α in dithiothreitol (DTT) (ER stress inducer) and morin hydrate-dithiothreitol (MH-DTT)-treated HepG2 cells at the maximum concentration of Mh (40 μM) for 24 h. Our results showed that treatment of MH-DTT for 24 h led to the significantly reduced expression levels of GRP-78, PERK and IRE1α with respect to control and DTT-treated HepG2 cells (Figure 2I,J).

**Figure 3:**
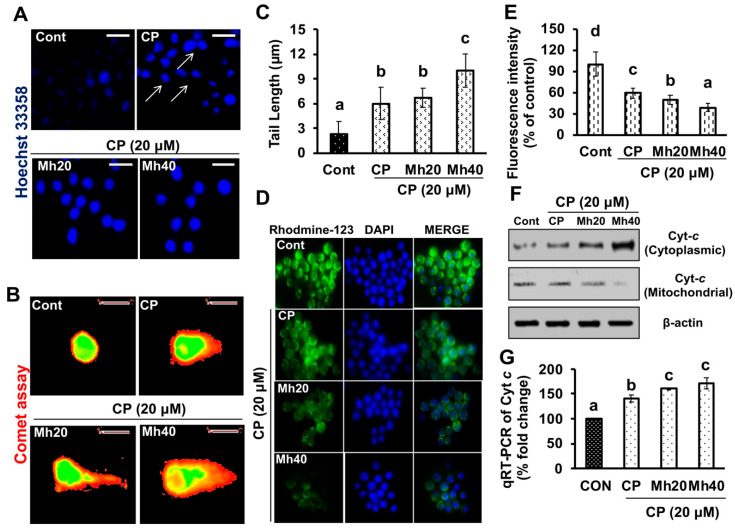
Effect of CP and CP-Mh on cellular apoptosis. (**A**) HepG2 cells were stained with the Hoechst-33342; condensed and fragmented nuclei indicated by arrows. (**B**) DNA damage evaluated by comet assay; images were captured by fluorescence microscope (magnification 400×, scale 0.1 mm). (**C**) Graphical representation of comet tail length. (**D**) Mitochondrial membrane potential disruption in HepG2 cells was assayed by Rhodamine-123 green stain after corresponding drug treatment and visualized by fluorescence microscopy (scale bar 0.1 mm). (**E**) Fluorescence intensity of Rhodamine-123 stain measured using ImageJ software. (**F**) Expression levels of the major mitochondrial apoptosis markers Cyt *c* was analyzed in the cytosolic and mitochondrial fraction proteins by Western blotting. β-actin was used as an internal control. (**G**) Quantitation of Cyt *c* mRNA level by quantitative real time-polymerase chain reaction (qRT-PCR) after corresponding drug treatment in HepG2 cells. The data are represented as the means standard deviation (±SD, *n* = 3). The values of different letters (a–d) differ significantly from each other (*p* < 0.05).

Next, to validate our previous findings underlying the cytotoxic activity of CP-Mh on HepG2^DR^ drug resistance cells, we examined the effect of CP-Mh on HepG2 cell death. First, we applied Hoechst 33358 stain to assess whether the combination of CP-Mh promotes apoptosis in HepG2 cells. As shown in Figure 3A, the number of pykno-nuclei formation increased in the CP-Mh-treated cells (bright blue color indicated by white arrow) in comparison to CP-treated and untreated HepG2 cells. In addition to this, we also performed comet assay to assess the single strand DNA breakage in CP and CP-Mh-treated HepG2 cells. As a result, an increased comet tail length was observed in CP-Mh-treated HepG2 cells with respect to CP-treated and control cells (Figure 3B,C). We further examined the effect of CP-Mh on the mitochondrial matrix potential (∆*Ψm*) using rhodamine-123. As shown in Figure 3D,E, a significant difference was observed in the rhodamine-123 fluorescence intensity in CP-Mh-treated HepG2 cells in comparison to CP-treated and untreated cells. Accumulating evidence indicates that loss of the mitochondrial membrane potential releases Cyt *c* that recruits pro-caspases to cleave into active caspases. The caspase cascade plays a regulatory role in cell apoptosis in various cancer cell lines. We, therefore, investigated the effect of CP-Mh on the Cyt *c* release from mitochondria to cytoplasm. As shown in Figure 3F, the level of the cytosolic Cyt *c* protein was markedly increased in CP-Mh-treated HepG2 cells with respect to CP-treated and control cells. However, the level of mitochondrial Cyt *c* was significantly reduced. Moreover, evaluation of the expression of Cyt *c* at the transcription level by real time-PCR showed an increased expression of Cyt *c* mRNA in CP-Mh-treated HepG2 cells, which was significantly less in CP-treated and untreated cells (Figure 3G). Current findings are consistent and validate our previous study published with HepG2^DR^ drug resistance cells [11].

### 2.3. Mh Promotes Apoptotic Cell Death in HepG2 Cells via JNK Pathway

**Figure 4:**
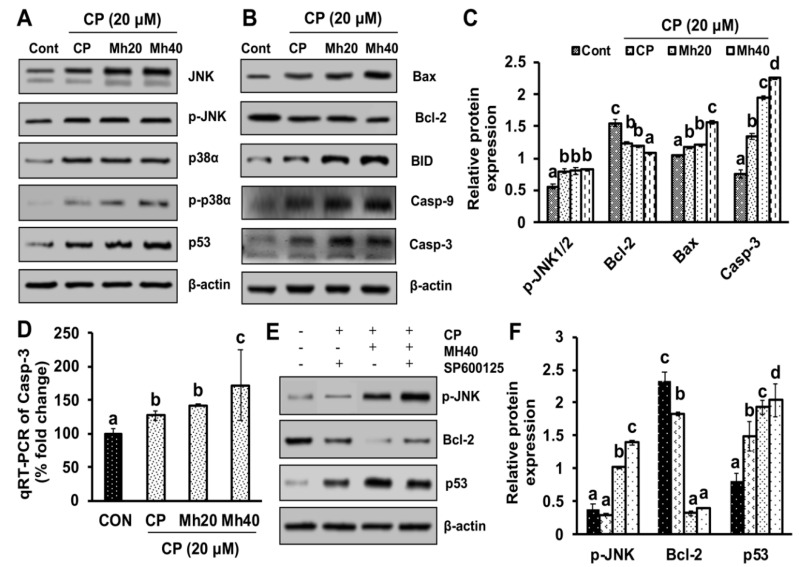
Apoptosis induction in HepG2 cells after CP and CP-Mh treatment. (**A**) Apoptosis signaling markers, including JNK, p-JNK, p38α, p-p38α and p53 were analyzed by Western blotting, results are normalized with β-actin. (**B**) Pro-apoptosis markers, including Bax, Bcl-2, BID, Casp-9 and Casp-3 were analyzed by Western blotting. Results are normalized to β-actin in respect to controls. (**C**) Relative protein expression of p-JNK, Bcl-2, Bax and Casp-3 analyzed by densitometry analysis using ImageJ software. (**D**) Quantitation of Casp-3 mRNA level by qRT-PCR after corresponding drug treatment in HepG2 cells. (**E**) Apoptosis signaling markers including p-JNK, Bcl-2 and p53 after corresponding drugs and SP600125 treatment were analyzed by Western blotting. Results are normalized with β-actin in respect to controls. (**F**) Relative expression of protein was calculated by densitometry analysis using ImageJ software. The data are represented as the means standard deviation (±SD, *n* = 3). The values of different letters (a–d) differ significantly from each other (*p* < 0.05).

To determine whether CP-induced ER stress activates the JNK signaling pathway of apoptosis, we examined the effect of Mh in combination with CP on the expression of JNK, p-JNK, p38α, p-P38α and p53 proteins. As shown in Figure 4A, significantly augmented expression levels of JNK, p-JNK, p38α, p-P38α and p53 were observed after CP-Mh treatment in HepG2 cells when compared with CP-treated and untreated cells. These results implicate that CP-Mh induced JNK-activation is an ER stress-independent phenomenon.

As the caspase cascade plays a regulatory role in the apoptosis of various cancer cell lines, we examined the effect of CP-Mh on the expression of key molecules of the apoptotic signaling cascade, including Bax/Bcl-2 ratio, BID, Casp-9 and Casp-3. As shown in Figure 4B,C, the expression levels of Bax/Bcl-2 ratio, BID, Casp-9 and pro/active-Casp-3 were increased significantly in CP-Mh-treated HepG2 cells when compared to CP-treated and control cells. However, the expression level of Bcl-2 was reduced in CP-Mh treated cells in a concentration-dependent manner. We further investigated the expression of Casp-3 at the transcription level by real time-PCR. An increased expression of Casp-3 mRNA was observed in CP-Mh-treated HepG2 cells, which was significantly less in CP-treated and untreated cells (Figure 4D). To prove whether the expression of pro-apoptotic signaling molecules is JNK-mediated, HepG2 cells were pre-treated with SP600125 (a JNK inhibitor, 10 μM) for 30 min, after that cells were exposed to different concentrations of CP and Mh. Results showed that SP600125 pre-treatment did not affect the expression level of JNK in CP-Mh-treated HepG2 cells; however, the expression levels of p53 and Casp-3, excluding Bcl-2, increased significantly in the CP-Mh-treated cells (Figure 4E,F).

### 2.4. CP-Mh Regulates PARP-1-Mediated Poly-ADP-Ribosylation and Subsequent HMGB1 Translocation in HepG2 Cells

**Figure 5:**
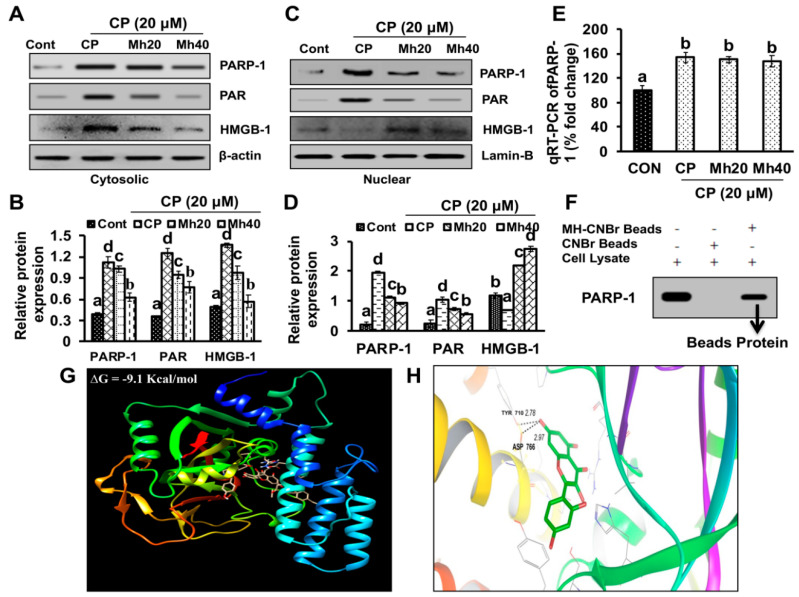
Regulatory effect of CP and CP-Mh on PARP-1-HMGB1 signaling. (**A**) The cytoplasmic expression of PARP-1, PAR and HMGB1 determined by Western blot analysis using specific antibodies after corresponding drugs treatment in HepG2 cells. Results are normalized to β-actin in respective controls. (**B**) Relative expression was calculated by densitometry analysis using the ImageJ software. (**C**) Nuclear expression of PARP-1, PAR and HMGB1 was analyzed using specific antibodies after corresponding drugs treatment in HepG2 cells. Results are normalized to Lamin B in respective controls. (**D**) Relative expression was measured by densitometry analysis using the ImageJ software. (**E**) Quantitation of PARP-1 mRNA level by qRT-PCR after corresponding drug treatment in HepG2 cells. The data are represented as the means standard deviation (±SD, *n* = 3). The values of different letters (a–d) differ significantly from each other (*p* < 0.05). (**F**) Binding of MH with PARP-1 in HepG2 cells detected by pull-down assay following Western blot analysis. (**G**) The interaction between MH and PARP-1 is shown in the docked complex; Mh is shown as stick model and PARP-1 is represented as ribbon model. (**H**) Hydrogen bonding and hydrophobic interaction between MH and PARP-1 calculated by shape complementary based algorithm.

To investigate the targeted effect of Mh on HepG2 cell death, we evaluated the expression level of PARP-1 following CP and CP-Mh treatment. Previous reports have proven that DNA damage-mediated PARP-1 activation plays the role in nucleo-cytoplasmic translocation of HMGB1 [17]. Cytoplasmic HMGB1 is a BECN-1 binding protein, which forms autophagosomes via poly-ADP-ribosylation by PARP-1 in cancer cells, which in turn contribute to reduced drug sensitivity through autophagy upregulation [9]. Therefore, we investigated the cytoplasmic and nuclear expressions of PARP-1, PAR and HMGB1 in CP and CP-Mh-treated HepG2 cells. As shown in Figure 5A–D, the amount of PARP-1, PAR and HMGB1 was significantly increased in the cytosolic contents of CP-treated HepG2 cells but was markedly reduced in CP-Mh-treated cells in a concentration-dependent manner with respect to control cells. However, the amount of nuclear PARP-1, PAR and HMGB1 was significantly reduced after CP-treatment, which was markedly increased in CP-Mh-treated cells with respect to control. Moreover, we also investigated the expression of PARP-1 at the transcription level using real time-PCR. We observed no significant changes in the PARP-1 mRNA expression after CP-Mh treatment in HepG2 cells when compared to CP treatment and control cells (Figure 5E).

Further, to validate our previous findings on whether Mh targets PARP-1, we performed the cell-based pull-down assay with cell lysates, using Mh-coupled cyanogen bromide (CNBr)-activated agarose beads. Endogenous PARP-1 in HepG2 cells lysates was pulled down by Mh-coupled CNBr-activated agarose beads (Figure 5F). Current findings support our previously published results that Mh inhibits the CP-induced HCC cell autophagy and activates HCC cell apoptosis by regulating PARP-1 [11]. Further, we employed *in-silico* analysis to validate the binding interactions between PARP-1 and Mh as per previous report [11]. The binding affinity of PARP-1 to Mh was predicted by docking. The program predicted the binding of Mh sites in the binding pocket of the human PARP-1. Pairwise alignment of Mh with Tyr 710 and Asp 766 amino acid of PARP-1 was observed (Figure 5G,H). The predicted binding affinity of Mh was estimated to be ∆G_bind_ = −9.1 kcal/mol.

### 2.5. CP-Mh Suppresses CP-Induced Autophagy Activation in HepG2 Cells

**Figure 6:**
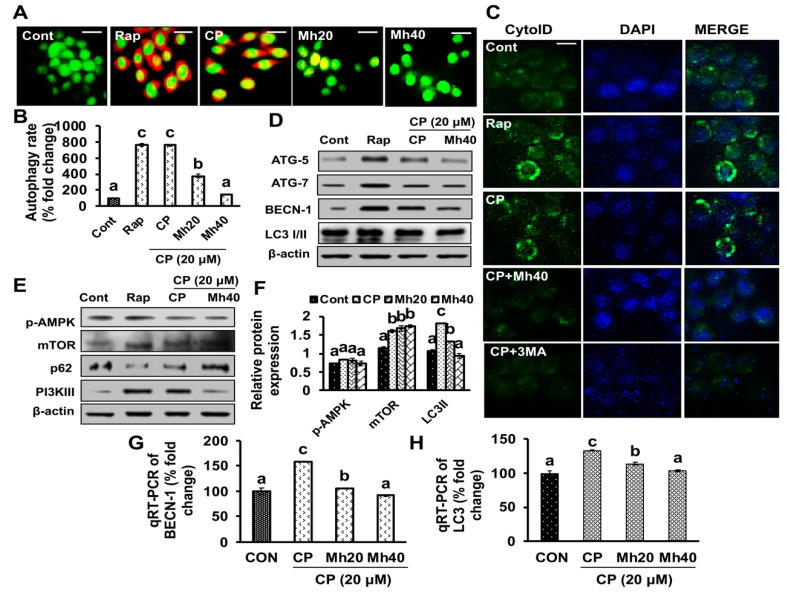
Effect of CP and CP-Mh on autophagy induction in HepG2 cells. (**A**) AO stained images of HepG2 cells taken after corresponding drug treatment by fluorescence microscopy; autophagosomes are visualized as red fluorescence (scale bar 0.1 mm). (**B**) Autophagy rate calculated manually using ImageJ software (**C**) Fluorescence images of HepG2 showing autophagic vesicles stained with Cyto-ID^®^ green visualized by fluorescence microscope. (**D**) Western blot analysis performed using specific antibodies for Atg5, Atg7, Beclin-1 and LC3I/II (**E**) Western blot analysis of p-AMPK, mTOR, p62 and PI3KIII after CP, CP-Mh and Rap treatment. (**F**) Relative expression of p-AMPK, mTOR and LC3I/II analyzed by densitometry analysis by ImageJ software. (**G**) Quantitation of BECN-1 mRNA level and (**H**) LC3 mRNA level by qRT-PCR after corresponding drug treatment in HepG2 cells. The data are represented as the means standard deviation (±SD, *n* = 3). The values of different letters (a–d) differ significantly from each other (*p* < 0.05).

To investigate the role of CP and CP-Mh in autophagy activation in HCC cell lines, the cells were stained with acridine orange (AO) dye. As shown in Figure 6A,B, CP-treated HepG2 cells were identified with bright red color accumulated in acidic vacuoles due to weak basic property of AO, which was monitored visually by a fluorescence microscope. However, 24 h treatment of CP-Mh resulted in a significant decrease in the percentage of red stained acidic vacuoles, when compared with CP, Rap-treated and untreated HepG2 cells. To further confirm the accumulation of acidic vacuoles, CytoID green fluorescence stain was used with DAPI as the background stain. The CP-treated HepG2 cells were identified as bright green color, which was monitored visually by fluorescence microscopy. However, the intensity of green fluorescence reduced significantly in CP-Mh and CP-3MA-treated cells, when compared with CP, Rap-treated and untreated cells (Figure 6C).

In a continuation of the fluorescence staining, we also assessed the expressions of various autophagy associated proteins such as PI3KIII, p-AMPK, mTOR, Atg5, Atg7, BECN-1, p62 and LC3I/II by Western blot analysis. As a result, the expression level of LC3I/II, a hallmark of autophagy induction, was increased significantly in CP and Rap-treated HepG2 cells. Consistent with the direct and indirect association of LC3I/II with p62, we next examined p62 expression after CP and Rap treatment, which was significantly reduced after 24 h in HepG2 cells. In contrast, the expression level of LC3I/II was significantly reduced in the CP-Mh-treated cells in comparison to CP, Rap and untreated cells. However, the expression level of p62 was increased in CP-Mh-treated cells. Moreover, the expression levels of related autophagy markers, such as PI3KIII, Atg5, Atg7 and BECN-1 were also significantly increased in CP and Rap-treated HepG2 cells and significantly reduced after treatment of CP-Mh. The expression of p-AMPK and mTOR was not altered significantly in CP, CP-Mh and Rap-treated HepG2 cells when compared with control (Figure 6D–F). Further evaluation in the expression levels of the major autophagy inducing markers (BECN-1 and LC3) at the transcription level revealed increased mRNA expression of BECN-1 and LC3 in CP-treated HepG2 cells, which was significantly reduced in CP-Mh-treated cells (Figure 6G,H). In addition, we also performed Western blot analysis to evaluate the expression levels of PARP-1 and LCI/II in Huh7, Hep3B and SK-Hep1 cells. As a result, a significant reduction in the expressions of above-mentioned autophagy markers was observed in CP-Mh-treated cells in a concentration-dependent manner when compared with CP-treated and untreated cells (Figure S2A–C). Above obtained results were consistent with our previous report published by Singh et al. 2019 [11].

### 2.6. CP-Mh Sensitizes HepG2 Cells to CP by Regulating PARP-1-HMGB1 Mediated Autophagy Activation

**Figure 7:**
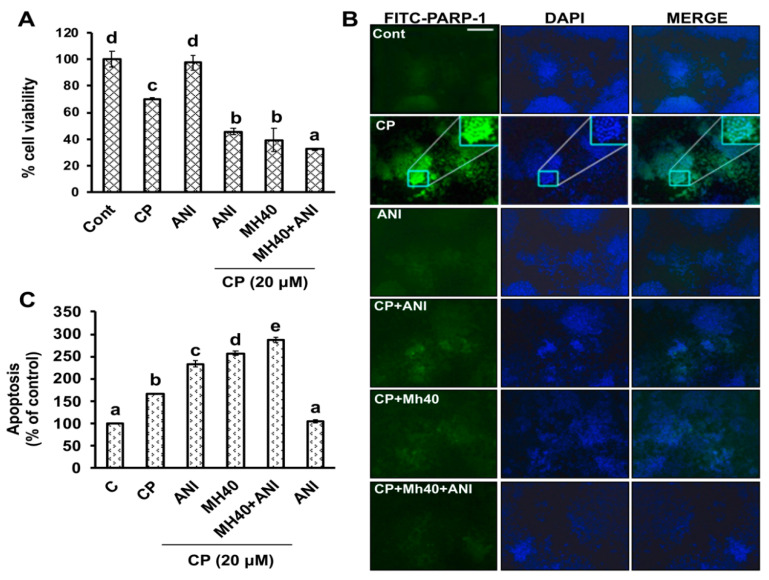
Inhibition of PARP-1 expression increases sensitivity of HepG2 cells to CP. (**A**) cell viability was evaluated by MTT assay after treatment with indicated concentrations of CP for 24 h in the presence or absence of ANI (10 μM) and MH (40 μM). (**B**) Immunocytochemistry of HepG2 cells for PARP-1 expression after corresponding drug treatment performed using fluorescein isothiocyanate (FITC) tagged anti-PARP-1 antibody and observed under fluorescence microscope (scale bar 0.1 mm); nuclear stain with DAPI (blue). (**C**) Percent apoptosis in HepG2 cells after corresponding drug treatment measured by ApoStrand™ enzyme-linked immunosorbent assay (ELISA) apoptosis detection kit. The data are presented as the means standard deviation (*n* = 3). Values with different letters (a–e) differ significantly from each other (*p* < 0.05).

On the basis of the known association between oxidative stress-induced PARP-1 activation and accumulation of unfolded proteins, we did a series of studies using HepG2 cells to determine the effect of CP in PARP-1-HMGB1-induced autophagy activation. To further validate our previous report with HepG2^DR^ drug resistance cells, the role of PARP-1 in autophagy induction and its inhibition to sensitize HepG2 cells towards CP, we compared the effect of Mh with synthetic PARP-1 inhibitor, 4-amino-1,8-naphthalimide (ANI). The cytotoxic effect of CP was evaluated with or without ANI (10 μM) by MTT assay. As shown in Figure 7A, CP, CP-Mh and CP-ANI-treated cells showed a significant reduction in HepG2 cells viability, while the cell viability of CP-Mh-treated cells was significantly less with respect to CP, CP-ANI-treated and untreated cells. Further, we performed fluorescence imaging to confirm the intracellular expression of PARP-1 by employing immunocytochemistry (ICC) assay. Interestingly, the distribution of overexpressed PARP-1 into strongly increased punctate structures appeared in CP-treated HepG2 cells, in contrast to treatment with CP-ANI, CP-Mh and ANI, where the expression of PARP-1 was significantly reduced (Figure 7B). In addition, we performed the apoptosis assay of HepG2 cells after corresponding drug treatments. As shown in Figure 7C, there was a significant amount of increase in the percent apoptosis in the CP-Mh and CP-ANI-treated cells with respect to ANI, CP and untreated cells. However, percent cell apoptosis was significantly greater in CP-Mh-treated cells as compared to CP-ANI which was consistent with our HepG2^DR^ drug resistance cells study [11].

**Figure 8:**
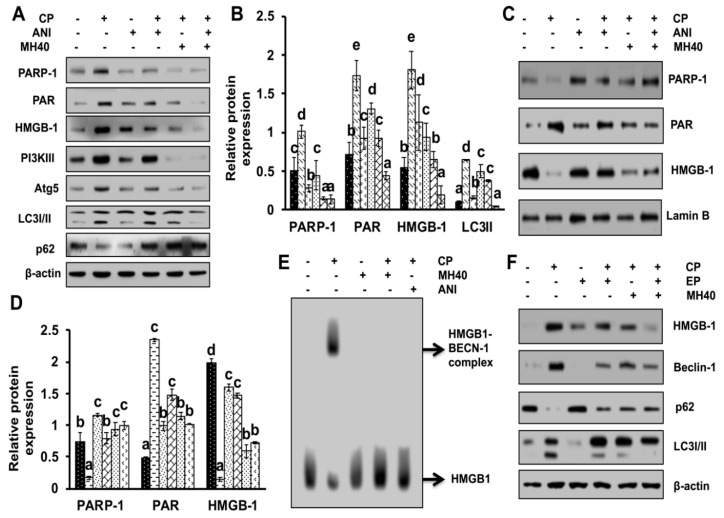
Effect of PARP-1 inhibitor ANI and MH on expression, translocation and release of HMGB1 in HepG2 cells. (**A**) Western blotting of cytosolic proteins was performed using specific antibodies against PARP-1, PAR, HMGB1, PI3KIII, Atg5, LC3I/II and p62 after treatment with CP, MH and ANI alone and in combination. Results are normalized with β-actin in respective controls. (**B**) Relative expression of PARP-1, PAR, HMGB1 and LC3I/II analyzed by densitometry analysis using ImageJ software. (**C**) The expression level of nuclear proteins was analyzed using antibodies against PARP-1, PAR and HMGB1 after treatment with CP, MH and ANI alone and in combination. Results are normalized to Lamin B in respective controls. (**D**) Relative expression of PARP-1, PAR and HMGB1 LC3I/II was measured by densitometry analysis using ImageJ software. (**E**) The HMGB1-Beclin1 interaction was evaluated by native gel after corresponding drug treatment. (**F**) The expression level of cytosolic proteins was analyzed using specific antibodies for HMGB1, Beclin-1, p62 and LC3I/II after treatment with CP, MH and EP alone and in combination. β-actin was used as an internal loading control. The data are presented as means standard deviation (*n* = 3). Values with different letters (a–e) differ significantly from each other (*p* < 0.05).

Since HMGB1 is required for PARP-1-regulated autophagy activation; we next examined the effect of CP-ANI and CP-Mh on the expression and translocation of HMGB1 in HepG2 cells. As shown in Figure 8A,B, the expression levels of different cytosolic proteins, such as PARP-1, PAR, HMGB1, PI3KIII, Atg5, LC3I/II and p62 were analyzed by Western blot analysis. We observed a significant reduction in the expression levels of PARP-1, PAR, HMGB1, PI3KIII, Atg5 and LC3I/II and an increased expression of p62 protein after CP-ANI and CP-Mh treatment, as compared to CP, ANI and untreated HepG2 cells. However, the expression level of PARP-1, PAR and HMGB1 significantly increased in nuclear proteins after CP-ANI and CP-Mh treatment, when compared with CP, ANI and untreated HepG2 cells (Figure 8C,D). Furthermore, to validate our previous study with HepG2^DR^ cells, the HMGB1-BECN-1 interaction in HepG2 cells after CP treatment was confirmed on native gel following Western blotting. As Figure 8E shows, two bands were observed with anti-HMGB1 (one at 90 kDa (bound HMGB1-BECN-1) and another at 29 kDa (free HMGB1) in CP-treated HepG2 cells; however, the CP-ANI and CP-Mh-treated cells showed only a single HMGB1 band at 29 kDa. These findings validate that Mh-mediated suppression of PARP-1 expression and cytoplasmic HMGB1 translocation are relatively safe ways to increase the sensitivity of CP in HepG2^DR^ and HepG2 cells by inhibiting HMGB1-BECN-1-mediated autophagophore formation [11]. Moreover, to confirm whether the cytosolic translocation of HMGB1 induces autophagy in CP-treated cells, we used ethyl EP (20 mM) to block the cytosolic translocation of HMGB1. As Figure 8F shows, the cytosolic expression of HMGB1 was reduced significantly in the CP-EP and CP-Mh-treated HepG2 cells when compared with CP, EP and untreated cells. In addition, the expression levels of Beclin1 and LC3I/II were significantly reduced and p62 level was significantly increased in the CP-EP and CP-Mh-treated cells.

**Figure 9:**
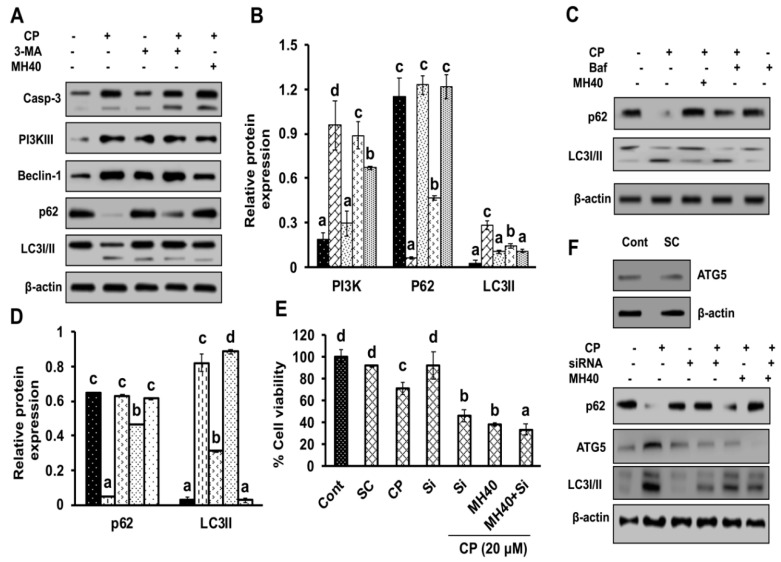
Inhibition of autophagy by 3-MA, baf, Atg5-siRNA and Mh enhances the sensitivity of HepG2 cells to CP. (**A**) Western blot analysis of autophagy and apoptotic markers was done using specific antibodies against Casp-3, PI3KIII, Beclin-1, p62 and LC3I/II after treatment with CP, MH and 3-MA alone and in combination. β-actin was used as an internal loading control. (**B**) Relative expression of PI3KIII, p62 and LC3I/II analyzed by densitometry analysis using ImageJ software. (**C**) The expression level of p62 and LC3I/II was analyzed after treatment with CP, MH and baf alone and in combination. Results are normalized to β-actin in respective controls. (**D**) Relative expression of p62 and LC3I/II analyzed by densitometry analysis using ImageJ software. (**E**) Cell viability of HepG2 cells was analyzed by MTT assay after treatment with CP, MH and Atg5-siRNA. (**F**) The expression level of p62, Atg5 and LC3I/II was analyzed after treatment with CP, MH and Atg5-siRNA alone and in combination. Results are normalized with β-actin in respective controls. The data are presented as the means standard deviation (*n* = 3). Values with different letters (a–d) differ significantly from each other (*p* < 0.05).

To confirm the role of autophagy suppression in HepG2 cells sensitivity, we used 3-MA (2.5 mM), bafilomycin (50 nM) and Atg5 siRNA as autophagy inhibitors and determined the expression levels of key autophagy and apoptosis markers such as Casp-3, PI3KIII, Atg5, p62 and LC3I/II. As shown in Figure 9A–D, the expression levels of PI3KIII, Atg5 and LC3I/II were significantly reduced in the HepG2 cells after 3-MA and bafilomycin treatment in combination to CP. However, the expressions of Casp-3 and p62 were increased with respect to CP-treated and control cells. Moreover, we observed that in comparison to CP alone, co-treatment of HepG2 cells with CP in combination with 3-MA, MH and baf, enhanced the cell cytotoxicity or apoptosis. In addition, by knocking down the expression of autophagy-related genes Atg5 with siRNA, the viability of HepG2 cells was significantly reduced in combination of CP with siRNA and Mh, in comparison to CP alone and control cells. However, the expression of Atg5 and LC3I/II was significantly suppressed after corresponding drug treatment, while there was an increase in the p62 expression (Figure 9E,F).

### 2.7. Mh-Mediated PARP-1 Regulation Increases Anti-Tumor Activity of CP in Hepatoma-Bearing Mice

**Figure 10:**
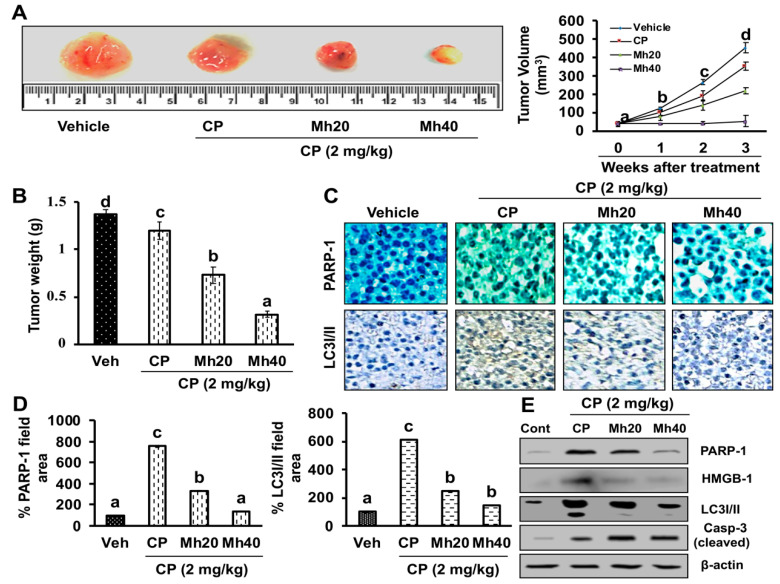
CP-Mh inhibits the growth of human hepatocellular carcinoma (HCC) xenograft in vivo. (**A**) Representative images of HepG2 xenograft nude mice display regression of tumor volume after treatment with CP-Mh in comparison to CP-treated and untreated groups. (**B**) Dissected tumors from vehicle CP and CP-Mh-treated xenograft mice; change in tumor weight observed after initiating the treatment. (**C**) The expression of PARP-1 and LC3I/II were determined by immunohistochemistry, (**D**) percentage of IHC stained proteins (PARP-1 and LC3I/II) were quantified by ImageJ. (**E**) Representative blots showing the total PARP-1, HMGB-1, LC3-I/II and Casp-3 in the vehicle, CP and CP-Mh-treated xenograft tissues (scale bar = 0.1 mm). The data are represented as mean standard deviation of three independent experiments (*n* = 3). Values with different letters (a–d) differ significantly from each other (*p* < 0.05).

To evaluate the antitumor effect of CP-MH in vivo, human HCC xenografts were established via subcutaneous injection of HepG2 cells into the lower right flanks of nude mice. After indicated amount of drug treatment, the body weights of the mice were monitored and recorded. As a result, no significant change in body weights of the CP and CP-Mh-treated groups were observed when compared to the vehicle-treated controls. However, CP-Mh treatment at a dose of 20 mg/kg and 40 mg/kg showed a significant inhibitory effect against tumor growth (tumor volume and weight) over time, with respect to the CP and vehicle-treated groups (Figure 10A,B). At the end of the last dose of treatment, the control and experimental mice were euthanized and the tumors were surgically removed for histological examination by immunohistochemistry (IHC) analysis of PARP-1 and LC3. IHC analysis of the tumors revealed reduced expression levels of PARP-1 and LC3 in the tumors treated with CP-Mh, whereas the CP-treated tumors showed much higher PARP-1 and LC3I/II expression (Figure 10C,D). These results were further validated by performing the immunoblotting of PARP-1, HMGB1, LC3I/II and Casp-3 markers in tumor tissue samples. Compared to the CP-treated group, the levels of PARP-1, HMGB1 and LC3I/II proteins were significantly reduced in the CP-Mh-treated groups, however, there was an increase in the expression level of Casp-3 (Figure 10E). Current findings are consistent with our previously published report with HepG2^DR^ xenograft model of mice [11].

## 3. Discussion

Hepatocellular carcinoma (HCC) is one of the leading causes of cancer-mediated deaths across the world. Although combinational therapy is considered to be the foremost method to restrict the invasion of metastatic disease, there are minimal chances of survival due to reduced chemosensitivity of cancerous cells. Therefore, to enhance the chemosensitivity towards cancer cells or tumor, conventional drugs are essential for improving the efficacy of available medications in clinical use [18]. The results of the present study show that CP-induced PARP-1 expression is capable of developing chemoresistance in HepG2 cells, which is facilitated through the HMGB1-mediated autophagy activation which also validate our previous report studied with HepG2^DR^ multi drug resistance cells [11]. Autophagy is a homeostatic cellular mechanism that plays a crucial role in response to metabolic and therapeutic stresses and attempts to sustain homeostatic balance through the lysosomal digestion of accumulated proteins and damaged organelles. Recent reports indicate that autophagy is a constitutive target towards chemotherapy, the induction of autophagy in response to chemotherapeutics is beheld as having a pro-death or a pro-survival role, thereby contributing to the anticancer sensitivity of these drugs as well as resistance [19]. Therefore, the combination of autophagy inhibitors and chemotherapeutic agents or molecular-targeted drugs is regarded as promising therapeutic strategy in the treatment of HCC. Autophagy is functionally activated in squamous cell carcinoma and HCC after cisplatin and oxaliplatin treatment and the suppression of autophagy by targeted inhibitors, such as 3-MA enhances apoptotic cell death [20,21]. The combined treatment of an autophagy inhibitor and bevacizumab markedly inhibits the growth of HCC; however, autophagy contributed in HCC cells survival [22]. Moreover, the combination of sorafenib and chloroquine generates ER stress-mediated cell death in HCC, both in vitro and in vivo [23]. In the present study, we used Mh to target PARP-1-mediated autophagy activation on CP treatment in HepG2 cells. Although the role of Mh as an inhibitor of LPS-induced autophagic cell death in RAW 264.7 cells has already been reported, the mechanisms need to be explored [24].

CP is a conventional platinum-based drug globally used to treat extremely progressive HCC. Even though responsiveness is high at the commencement, in due course, the cells start evolving resistance towards these drugs. Emerging evidences have demonstrated that PARP-1 overexpression leads to chemoresistance to cisplatin in non-small cell lung carcinoma [25]. However, inhibition of PARP-1 expression induces increased sensitivity towards the cell death in breast cancer cells [26]. Moreover, it has also been shown that PARP-1 is capable of poly-ADP-ribosylation of HMGB1 to induce chemoresistance through autophagy activation in Jurkat cells (human T lymphocyte cells), mouse embryonic fibroblast cells and HepG2^DR^ cells [11]. These cells were sensitized towards drugs by 4-amino-1, 8-naphthalimide and ethyl pyruvate. These studies determine that endogenous PARP-1 induces innumerable factors in cancer cells which account for the development of chemo-resistance [11,27,28]. In our study, we demonstrate that HepG2 cells were able to sensitize against CP in the presence of Mh by targeting PARP-1. We also found that a PARP-1 inhibitor such as ANI could sensitize the HepG2 cells towards CP treatment, which was comparable with Mh. This suggests that the increase in the level of PARP-1 can develop chemoresistance in HepG2 cells and confirms the role of PARP-1 in chemo-resistance, similar to our previous findings [11].

Previous studies have reported that autophagy reduces the sensitivity of chemotherapeutic agents against cancer cells by decreasing the apoptotic potential of drugs [19]. Although the molecular mechanism of developing chemoresistance by autophagy is as yet unclear, this has led autophagy to be a fundamental target to disrupt the chemoresistance in cancer cells. Studies have demonstrated that chemically induced excessive oxidative stress leads to DNA damage and further PARP-1 activation, consequently as a positive feedback mechanism that exacerbates necrotic cell death [19]. PARP-1 activation following DNA damage results in the PARylation of several proteins, including PARP-1. In addition, PARP-1 is involved in autophagy activation, thus promoting cell survival [11,29]. CP-induced upregulation of the full-length PARP-1 correlated well with PAR formation, while the PARP-1 inhibitor ANI enhanced CP-induced cell death. The roles of PARP-1 inhibitors such as ANI and 3-AB in cancer chemotherapy have been reported previously [11,29]. PARylated PARP-1 is accompanied by obvious upregulation of HMGB1 and LC3II in breast cancer cells [28,30]. In this study, we observed significant development of ROS, DNA damage and induced expression of DNA damage associated protein such as H2AX and PARP-1. In addition, we also observed significant increase in comet tail and comet head diameter of CP-treated HepG2 cells. Moreover, we demonstrated that the induced PARP-1 expression increased the expression of LC3II in the progression of autophagy. However, these CP-induced changes in parameters were significantly mitigated after Mh treatment in a concentration-dependent manner in HepG2 cells, which progressed to apoptotic cell death. On the basis of current findings, we propose that PARP-1 is able to induce autophagic flux in response to CP treatment. However, the signaling mechanism needs to be elaborated in detail.

Previous researches have suggested that the cytosolic release of Cyt *c* during apoptosis is sought to occur through the increase mitochondrial permeability, swelling and rupture of the mitochondrial outer membrane due to loss of mitochondrial membrane potential by chemotherapeutic drugs [31]. It has further been reported that cisplatin-based chemotherapies induce apoptosis by reducing the mitochondrial membrane potential in ovarian cancer cells [32]. Conversely, cancer cells have developed strategies like autophagy to protect drug-induced mitochondrial damage. A recent report has published that, in ovarian cells, increased autophagy activation diminishes mitochondrial apoptosis and membrane potential loss induced by CP [33]. However, inhibition of autophagy with specific inhibitors and siRNA in cisplatin-treated ovarian cancer cells has augmented apoptosis through the mitochondrial signaling pathway [33]. Above mentioned studies suggested that CP-induced autophagy is capable of inhibiting the mitochondrial membrane potential loss which provides chemoresistance towards certain types of cancers. In our previous and current study, we did find loss of mitochondrial membrane potential in CP-treated hepatoma cells with respect to control; this loss of MMP was significantly increased after combination treatment of CP and Mh [29]. Therefore, the results indicate that the PARP-1-triggered autophagy helps to suppress mitochondrial apoptosis and develops resistance to CP towards HepG2 cells. However, our results suggest that adjuvant effect of Mh with CP induces cell death in HepG2 cells, which is mediated by the JNK signaling pathway of apoptosis.

According to previous reports, chemical-induced oxidative stress leads to the development of ROS, which subsequently induces PARP-1 expression; the role of PARP-1 in Ca^++^ efflux through endoplasmic reticulum (ER) has been evidenced in several studies [34]. ER facilitates the proper proteins folding and serves as an intracellular Ca^++^ store. ER stress appears in response to different physiological and pathological conditions, such as protein aggregation, limited protein glycosylation, lack of glucose and hypoxia [35]. In these situations, a specific unfold protein response (UPR) pathway of nuclear signaling is activated, that results in the regulation of global protein synthesis and leads to increased production of proteins required for proper folding at ER, such as chaperones. GRP78 is a cytoprotective protein overexpressed by ER stress causing factors, such as unfolded protein response (UPR), intracellular Ca^++^ efflux, glucose deprivation and pathological conditions [36]. However, the up-regulation of GRP78 protein promotes cell survival; in that way helps the normal cells in tissue preservation and organ fortification. Conversely, it is also believed to cause immune resistance, induce tumor progression, malignancy and drug-resistance [37]. In the present study, we found increased intracellular ROS production and Ca^++^ release in CP-treated HepG2 cells. Moreover, the expression of IRE1α, calnexin and GRP78 was also increased. However, the expression of the above-stated parameters was significantly ameliorated in a concentration-dependent manner in CP-Mh-treated HepG2 cells. On the basis of our findings, we suggest that ER-stress positively regulates autophagy activation induced by CP treatment in HepG2 cells. We also suggest that in an oxidant milieu, co-treatment of Mh and CP creates an anti-oxidant environment that protects the host from the prevalence of HepG2 cells.

**Figure 11:**
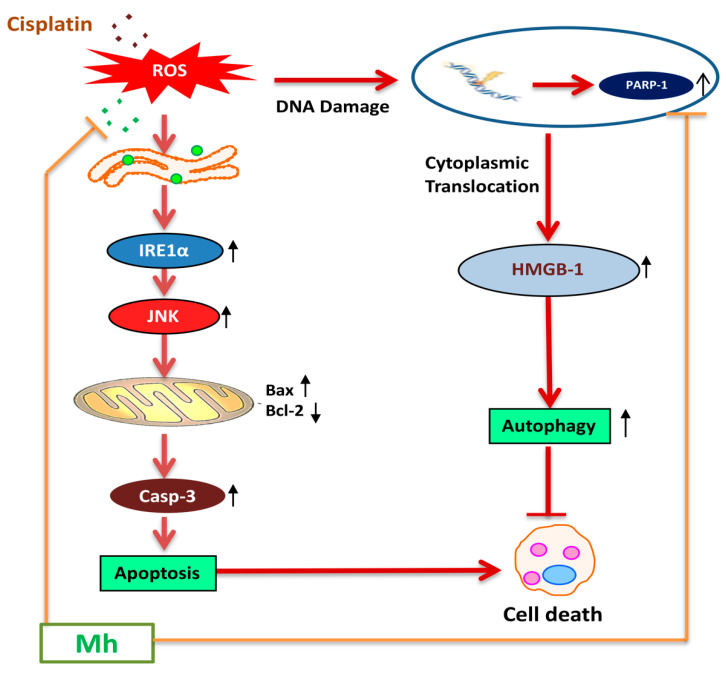
Schematic representation of mechanisms of CP-Mh regulated apoptosis and autophagic in vitro and in vivo. Cisplatin induced PARP-1 leads to HMGB1 mediated autophagy activation and consequently inhibited apoptosis. Combination of CP-Mh reduced autophagy activation via regulating ROS generation and PARP-1 expression and sensitized HCC cells towards JNK-mediated apoptotic cell death.

To validate whether CP-induced PARP-1 develops autophagy-mediated chemo-resistance in HepG2 cells, we found PARP-1 to be the key molecule that induces HMGB1-mediated autophagy activation [11]. It has been demonstrated that autophagic stimuli promote HMGB1 translocation and direct interaction with Beclin1 in the cytosol to induce autophagy [9]. In our data, the downstream signals of PARP-1-HMGB1 pathway, such as PAR, BECN-1, Atg5 and LC3I/II in the cytosolic protein, were up-regulated in CP-treated HepG2 cells. The expression of these major autophagy inducing proteins was attenuated in HepG2 cells during co-treatment with CP plus a higher concentration of Mh; suppression of PARP-1 also inhibited CP-induced HMGB1-BECN-1 complex formation as well as autophagy activation which was also consistent with our previous report [11]. We further did a comparative study with ANI (a PARP-1 inhibitor) and EP (HMGB1 inhibitor). In addition, co-treatment of CP with Mh, 3-MA, baf and Atg5-siRNA mitigated the autophagy activation and induced HepG2 cell death. Above stated results indicate that CP-induced upstream PARP-1 activation plays a pivotal role in downstream HMGB1, PAR, p62, Atg5 and LC3I/II activation, which plays a protective role in HCC subjected to cytotoxicity of CP [38]. In contrast, our findings validate that Mh plays a death-promoting role in HepG2^DR^ and HepG2 cells via PARP-1-regulated inhibition of autophagy [11,39]. Furthermore, in the hepatoma xenograft model, CP-Mh pre-treatment inhibited tumor growth and tumor weight. Translational expression analysis confirmed the decrease in PARP-1, HMGB1 and LC3I/II levels following the combinational treatment of CP-Mh, revealing that Mh exerts a therapeutic efficacy against HCC in the combination of CP chemotherapy. A detailed schematic summary of plausible mechanisms of CP-Mh regulated apoptosis and autophagic cascade has been illustrated in Figure 11.

## 4. Materials and Methods

### 4.1. Chemicals

Morin hydrate (Mh), cisplatin (CP), DMEM, 3-(4,5-dimethylthiazol-2-yl)-2,5-diphenyltetra-zolium bromide (MTT), acridine orange (AO), Hoechst-33342, 4′-6-diamidino-2-phenylindole (DAPI), JC-1, 2′,7′-dichloro-fluorescin diacetate (H_2_DCFDA), fura-2AM, Rhodamine-123, rapamycin (rap), dithiothreitol (DTT), ethyl pyruvate (EP), SP600125, 4-amino-1,8-naphthalimide (ANI), 3-methyl adenine (3-MA) and bafilomycin, were supplied by Sigma-Aldrich, St. Louis, MO, USA. Fetal bovine serum (FBS) was delivered by Gibco (Gaithersburg, MD, USA). The information of antibodies used in the present study has been provided in the Supplementary Table S1.

### 4.2. Cell Culture and Treatment

HepG2, Huh7, Hep3B and SK-Hep1 human liver hepatoma cells were purchased from ATCC and cultured in DMEM media with 10% heat-inactivated FBS and 1% penicillin/streptomycin in a CO_2_ incubator at 37 °C. 1 × 10^5^ cells/well were cultured in 12-wells microtiter plates and treated with varying concentrations of CP (20 μM) and Mh (20 and 40 μM) for 24 h; cells were then centrifuged (600*g* × 3 min) and harvested. All the drugs were solubilized in 100% dimethyl sulfoxide (DMSO) (with final concentration of 0.1%).

### 4.3. MTT Bioassay

The 3-(4,5-dimethylthiazol-2-yl)-2,5-diphenyltetrazolium bromide (MTT) assay is also known as cell viability assay, which measures the metabolic status of cells, particularly in mitochondria by reflecting early cellular redox changes. Briefly, cells (1 × 10^5^ cells) were seeded in a 96-well plate and incubated with CP, CP-Mh and Mh alone (20 and 40 μM) for 24 h in a CO_2_ incubator at 37 °C. Afterwards, the cells were exposed to MTT (5 mg/mL) for 4 h. The dark-blue formazan crystals were dissolved in 100% DMSO and optical density was measured at 540 nm using an ELISA plate reader (Bio-Tek Instrument Co., Winooski, WA, USA). The percent cell viability was calculated with respect to control by the following formula.

Cell viability (%) = (Treated group/control group) × 100.(1)

### 4.4. Lactate Dehydrogenase Assay

Intra- and extra-cellular activities of lactate dehydrogenase (LDH) in CP, Mh and CP-Mh-treated HepG2 cells were measured spectrophotometrically. Cytotoxicity Detection Kit (Sigma-Aldrich, St. Louis, MO, USA) was used following the manufacturer’s instructions.

### 4.5. Evaluation of Cell Morphology

HepG2 cell morphological assessment was performed following post-exposure of CP, Mh and CP-Mh for 24 h at 37 °C. For that purpose, cells were visualized under a compound microscope (Nikon Eclipse TS200, Nikon Corp., Tokyo, Japan) at 200× magnification.

### 4.6. Apoptotic Cell Death Assay

Since the DNA present in the cell nucleus, showing apoptotic characteristics, is sensitive to formamide exposure. This denatured DNA can be detected using a monoclonal antibody against single-stranded (ss) DNA provided by ApoStrand™ enzyme-linked immunosorbent assay (ELISA) apoptosis detection kit (Enzo Life Sciences, Plymouth Meeting, PA, USA), following the manufacturer’s protocol.

### 4.7. Clonogenic Assay

Clonogenic assay was performed to evaluate the cytotoxicity of CP and CP-Mh. Briefly, HepG2 cells (300 cells/well) were seeded in 6-well tissue culture plates (BD Falcon, Sandiego, CA, USA) and allowed to attach overnight. Afterwards, the cells were exposed to various concentrations of corresponding drugs for 24 h. Then the cells were washed in phosphate buffer saline (PBS) and re-seeded in fresh medium till 10 days to form colonies. Cells were subsequently washed with PBS, fixed with methanol/acetic acid (3:1 ratio) solution and stained with 0.1% (*w*/*v*) crystal violet dye for 10 min. The stained colonies were rinsed with tap water and, counted manually using the ImageJ software (www.rsbweb.nih.gov/ij/).

### 4.8. Acridine Orange (AO) Staining

Acidic intracellular compartments formed during autophagy activation were visualized by acridine orange (AO) staining. Briefly, CP and CP-Mh exposed HepG2 cells were washed with PBS and stained with AO (10 μg/mL) (Sigma-Aldrich, St. Louis, MO, USA) for 15 min at 37 °C. Cells were then rinsed with PBS and observed under a fluorescence microscope at 200× magnification.

### 4.9. Cyto-ID Green Fluorescence Staining for Autophagy Detection

CytoID green fluorescence dye was used to specify the location of autophagic vacuoles in HepG2 cells, for this, cells were grown on a cell culture cover glass (SPL, Seoul, Korea). After that cells were pre-treated with 3-MA (2.5 mM) for 1 h, followed by exposure to various concentrations of corresponding drugs for 24 h at 37 °C. Cells were then fixed with 4% paraformaldehyde for 10 min. Cells were stained with Cyto-ID Green Detection Reagent (Enzo Life Sciences, Plymouth Meeting, PA, USA) and visualized using fluorescence microscope as per the manufacturer’s instructions. Briefly, cells were gently washed with PBS and stained for 30 min with Cyto-ID green dye prepared in an indicator-free medium with 5% FBS and incubated at 37 °C. Hoechst 33342 was used as a counter stain to stain the nucleus. The cover slips were rinsed with assay buffer and observed under a fluorescence microscope at 200× magnification.

### 4.10. Mitochondrial Membrane Potential (MMP) Assay

Mitochondrial membrane potential (MMP) assay was performed as described previously [11]. MMP (∆Ψm) was monitored by fluorescence dye. HepG2 cells were cultured on glass coverslips in 12-well plates following treatment with various concentrations of drugs for 24 h at 37 °C. The cells were then rinsed with PBS and stained with the Rhodamine-123 dye (1 μg/mL). Afterwards, cells were visualized by fluorescence microscope at 400× magnification.

### 4.11. Detection of Intracellular Reactive Oxygen Species (ROS)

ROS generation was detected by the method described previously [11]. For the detection of intracellular ROS generation in HepG2 cells, cells were treated with various concentrations of corresponding drugs for 24 h at 37 °C, rinsed with PBS, exposed to 10 µM peroxide-sensitive fluorescent probe H_2_DCFDA for 30 min at 37 °C. Cells were then analyzed for ROS production with a fluorescence microscope at 400× magnification.

### 4.12. Detection of Ca^++^ Production

Ca^++^ measurement was performed as described previously [11]. HepG2 cells was exposed to various concentrations of CP and CP-Mh. Cells were then immersed in Fura 2-AM (5 μM) dye for 60 min at 37 °C and dissolved in Hank’s balanced salt solution (HBSS) buffer. Cells were then rinsed 3x with HBSS buffer to remove extracellular dye [40]. Stained cells were visualized under fluorescence microscope at 400× magnification.

### 4.13. Sub-Cellular Fractionation

Cytosolic and nuclear fractions of cells were isolated by the method described previously [11], using an NE-PER nuclear protein extraction kit (Thermo Scientific, Seoul, Korea), according to the manufacturer’s protocol.

### 4.14. Isolation of Mitochondrial Protein

The method of preparation of mitochondrial-enriched fractions was described in our previous research [11]. Cells were pelleted by centrifugation, re-suspended in mitochondrial extraction buffer. Cells were homogenized using a Dounce homogenizer and centrifuged at 600× *g* for 10 min. The resulting pellet was discarded and the supernatant was collected into a fresh Eppendorf tube; and centrifuged again at high speed (11,000× *g*, 10 min) to separate mitochondria from soluble cytosolic contents.

### 4.15. Immunoblotting

Cells were harvested and re-suspended in radioimmunoprecipitation assay (RIPA) lysis buffer (Sigma, St. Louis, MO, USA) to isolate the total fraction of protein. The amount of protein was quantified by Bradford assay. An equal amount of protein (30 μg/well) was loaded to SDS–polyacrylamide gel electrophoresis or native-PAGE (for interaction study) to perform Western blot analysis. Protein from sodium dodecyl sulfate polyacrylamide gel electrophoresis (SDS-PAGE) was transferred to a polyvinyldenefluoride (PVDF) membrane (Roche Diagnostics, Indianapolis, IN, USA) by electroplating. The blots were exposed to primary antibodies for minimum 4–5 h, followed by horseradish peroxidase-conjugated secondary antibody for 2 h. Afterwards, blots were developed by using enhanced chemiluminescence (ECL), as per recommended protocol (Amersham Pharmacia, Piscataway, NJ, USA).

### 4.16. Detection of Nuclear DNA Condensation with Hoechst-33342 Stain

To detect the morphologic features of chromatin, HepG2 cells were stained with Hoechst- 33342 dye according to Diaz-Ruiz et al. [41]. Briefly, cells were seeded in a 96 well plate, treated with desired concentrations of CP and CP-Mh for 24 h at 37 °C. Cells were then washed 2× with sterile PBS and fixed by using 4% formaldehyde. Cells were rinsed again with PBS and exposed to Hoechst-33342 (1 µg/mL) for 10 min at 37 °C. Cells were washed with PBS to remove the extra stain and observed under a fluorescence microscope at 400× magnification.

### 4.17. Comet Assay

To determine the intracellular DNA damage by comet assay, cells were treated, harvested and washed 3x with PBS and followed the procedure described by Singh et al. [42] with slight modifications. Briefly, normal glass slides were covered with 1% melting agarose dissolved in PBS and kept in 4 °C to solidify. Cells were suspended in 85 µL of low-melting agarose and layered onto the solidified agarose containing slide and left on ice for 10 min. The coated slides were immersed in cold lysis buffer (0.1 M Na_2_EDTA, 1% Triton X-100, 2.5 mM NaCl, 10 mM Tris and 10% DMSO, pH 10) for 1 h. After that slides were placed in alkaline buffer (0.3 M NaOH and 1 mM Na_2_EDTA, pH 13) for 25 min to allow DNA unwinding. Then electrophoresed for 30 min at 20 V adjusted at 300 mA. Finally, slides were gently immerged in neutralization buffer (0.4 M Tris, pH 7.5) for 25 min and apply 20 µg/mL of ethidium bromide. The relative amount of damaged DNA was observed under fluorescence microscope at 400× magnification.

### 4.18. Real-Time PCR

HepG2 cells were treated with CP and CP-Mh and RNA was isolated using RNA-spin™ Total RNA Extraction Kit (Intron Biotechnology, Seoul, Korea), following the manufacturer’s instructions. RNA was quantified using a Qubit22^®^ 2.0 fluorometer RNA Assay Kit (Life technologies, USA). Maxime RT Premix cDNA Synthesis Kit (Intron Biotechnology, Korea) was used to prepare cDNA. β-actin gene was also amplified under the same reverse transcription polymerase chain reaction (RT-PCR) parameters to normalize the quantitative data. Quantitative RT-PCR amplification was executed in Agilent Technology quantitative polymerase chain reaction (qPCR) System (Foster, CA, USA). Detailed list of the primers used for Cyt *c*, Casp-3, PARP-1, Beclin-1 and LC3 amplification is stated in Table S2.

### 4.19. Immunocytological Analysis

Immunocytological staining was performed according to the method described previously [11]. Cells were grown on a glass cover slip (SPL, Seoul, Korea) for 24 h, treated with indicated amount of drug. Cells were washed, fixed (4% paraformaldehyde in PBS) for 20 min and blocked with 3% normal goat serum. Afterwards, incubated with PARP-1 primary antibody for overnight at 4 °C. Next day, cells were washed and applied fluorescein isothiocyanate (FITC)-labeled goat anti-rabbit IgG secondary antibody for 1 h. The coverslips were washed with PBS, mounted with DAPI anti-fade mounting medium and visualized under a fluorescence microscope at 200× magnification.

### 4.20. siRNA Transfection

Cells were seeded and transfected with siRNA (25 pM) against Atg5 and negative control (Integrated DNA Technologies, Maryland, MD, USA), using 7.5 μL of lipofectamine^®^ RNAiMAX transfection reagent (Invitrogen, Maryland, MD, USA) in a 6-wells microtiter plate followed by 24 h of CP and CP-Mh treatment. Afterwards, the cells were lysed to extract proteins and expression levels of different autophagic markers, including β-actin were determined by Western blot analysis. The sequence of siRNA used for Atg5 knock down is mentioned in Table S3.

### 4.21. Protein-Ligand Interaction In-Silico

The 3D structure of PARP-1 was obtained from the Protein Data Bank (www.rcsb.org) (PBD ID: 5DS3), whereas the structure of Mh was retrieved from the ZINC database (ZINC03881558). The protein-ligand binding of PARP-1 and Mh was predicted by using Autodock (Hex v8.0.0 CUDA), followed by a visualization of the interaction using PyMol (ver 4.0). Protein residue sharing hydrogen and hydrophobic interactions with ligand molecules were calculated by shape complementary-based algorithm.

### 4.22. Cell-Based Pull-Down Assay

Mh was coupled with CNBr-activated agarose beads as per the manufacturer’s suggested protocol. Proteins (500 μg) from HepG2 cells and morin-coupled beads (100 μL) were mixed in a reaction buffer (150 mM NaCl, 1 mM phenylmethylsulfonyl fluoride (PMSF), 50 mM Tris (pH 7.5), 5 mM ethylenediaminetetraacetic acid (EDTA), 0.01% NP-40, 2 μg/mL bovine serum albumin (BSA), 1% protease inhibitor cocktail) and allowed to incubate with gentle rocking for overnight at 4 °C. After incubation, the beads were washed (5x) with washing buffer [5 mM EDTA, 50 mM Tris (pH 7.5), 1 mM PMSF, 150 mM NaCl, 0.01% NP-40] and binding was analyzed using Western blotting [43].

### 4.23. Antitumor Effect of CP-Mh In Vivo

Four-week aged male BALB/c-nu nude mice (15 ± 5 g) were supplied by Oriental Biotechnology (Seoul, Korea). All the animals were kept under specific germ-free environment with *ad libitum* food and water supply in the Laboratory Animal Care of Daegu University (LMO No. LML-16-1134). Experiments were performed according to the guidelines of Care and Use of Laboratory Animals of the National Institutes of Health, which were approved by the Committee on the Ethics of Animal Experiments of the Daegu University, South Korea.

HepG2 cells were cultured, harvested and re-suspended in sterile PBS. A total of five million cells (5 × 10^6^) were injected into the right flank of the animals subcutaneously. We used following formula: V= (L × W^2^)/2 to measure the volume of the tumor, in this formula, L stands for the length and W for width of the tumor nodules measured with Vernier calipers. Once the tumors volume reached 25–50 mm^3^, the mice were separated into four different groups (each n = 5). Every twice a week for 3 weeks, mice were injected (IP) of vehicle (0.1% DMSO, control group), CP (2 mg/kg body weight), CP-Mh20 (2 mg/kg–20 mg/kg body weight) and CP-Mh40 (2 mg/kg–40 mg/kg body weight). Body weights of mice were measured before each injection of drug. On the 21th day, the mice were sacrificed; the tumors were taken away, weighed and photographed.

To determine the antitumor effect of CP-Mh in vivo, histological analysis of hepatoma xenografts was performed. Briefly, the tissues were fixed in a 10% formalin solution for 24 h, processed with graded volumes of ethanol, embedded in paraffin, sectioned and stained for immunohistochemistry (IHC) using antibodies against PARP-1 and LC3I/II. The IHC images were taken under a light microscope at 200× magnification. The immune-reactive areas were quantified manually by the ImageJ software.

### 4.24. Statistical Analysis

SPSS (Statistical Package for the Social Sciences) 22.0 was used for statistical analysis. All the results were expressed as the mean ± standard deviation (SD) of three independent experiments. The data were analyzed using one-way analysis of variance (ANOVA), the significance difference between samples was considered statistically significant for values of *p* < 0.05, calculated by Duncan’s multiple range test (SPSS Inc., Chicago, IL, USA). Significant differences among the groups are indicated by the letters a, b, c, d & e, where, a, b, c, d, and, e represent *p* > 0.05, *p* < 0.05, *p* < 0.01, *p* < 0.001 and *p* < 0.0001, respectively.

## 5. Conclusions

In summary, we believe that our previous, as well as our current, studies are pioneers in demonstrating the regulatory effect of Mh against PARP-1-HMGB1-mediated autophagy activation and lead to modulation of CP sensitivity both in vitro and in vivo. The regulation of PARP-1-mediated autophagy consequently enhances the sensitivity towards this chemotherapeutic drug via apoptosis induction. These findings may help to expand the knowledge of autophagy pathways and provide a new vision for researchers towards how autophagy is regulated in HCC. Thus, we propose that PARP-1 should be explored with more experimental details as a therapeutic target for preventing the development of chemoresistance in HCC and for reinforcing the efficacy of CP against malignancy.

## Supplementary Materials

Supplementary materials can be found at https://www.mdpi.com/1422-0067/21/21/8253/s1. Supplementary materials include three tables (Tables S1–S3) and one figures (Figures S1 and S2). Table S1: Antibodies used in this study; Table S2: Sequences of RT-PCR oligonucleotide primers for this study; Table S3: Sequences of siRNA; Figure S1: Cytotoxic effect of CP and CP-Mh on hepatoma cells; Figure S2: Regulatory effect of CP and CP-Mh on PARP-1/LC3I/II protein expression.
