# Supplementary material for: Morin Hydrate Sensitizes Hepatoma Cells and Xenograft Tumor towards Cisplatin by Downregulating PARP-1-HMGB1 Mediated Autophagy
Source: Int J Mol Sci. 2020 Nov 4;21(21):8253. doi: 10.3390/ijms21218253 (PMC7885522; doi:10.3390/ijms21218253)
Supplement: Supplementary file 1 [file ijms-21-08253-s001.zip › ijms-983790-supplementary.docx]

**Supplementary Information**

**Morin hydrate sensitizes hepatoma cells and xenograft tumor towards cisplatin by downregulating PARP-1-HMGB1 mediated autophagy**

Mahendra Pal Singh ^1,2^, Tejinder Pal Khaket ^3^, Vivek K. Bajpai ^4^, Saleh Alfarraj ^5^, Se-Gie Kim ^6^, Lei Chen ^7,*^, Yun Suk Huh ^8^, Young-Kyu Han ^4,*^, Sun Chul Kang ^1,*^

^1^ Department of Biotechnology, Daegu University, Gyeongsan, Gyeongbuk, 38453, Republic of Korea

^2^ Department of Immunology, Mayo Clinic, Rochester, MN-55905, United States

^3^ Department of Radiation Oncology, The Ohio State University, Columbus, OH-43210, United States

^4^ Department of Energy and Materials Engineering, Dongguk University-Seoul, 30 Pildong-ro 1-gil, Seoul 04620, Republic of Korea

^5^ Zoology Department, College of Science, King Saud University, Riyadh 11451, Saudi Arabia

^6^ Department of Pharmaceutical Engineering, Daegu Catholic University, Gyeongsan, Gyeongbuk, 38430, Republic of Korea

^7^ College of Food Science, Fujian Agriculture and Forestry University, Fuzhou, Fujian 350002, China

^8^ Department of Biological Engineering, Biohybrid Systems Research Center (BSRC), Inha University, 100 Inha-ro, Nam-gu, Incheon 22212, Republic of Korea

**Corresponding authors***

[chenlei841114@hotmail.com](mailto:chenlei841114@hotmail.com) (L. Chen); E-mail: [ykenergy@dongguk.edu](mailto:ykenergy@dongguk.edu) (Y-.K. Han); [sckang@daegu.ac.kr](mailto:sckang@daegu.ac.kr) (S.C. Kang)

**Table S1.** List of antibodies used for Western blot analysis.

| Antibodies | Dilution used | Source | Catalog no. |
| --- | --- | --- | --- |
| **Primary antibodies** | | | |
| β-actin | 1:1000 | Santa Cruz Biotechnology | sc-1616 |
| Glutathione reductase | 1:1000 | Santa Cruz Biotechnology | sc-32886 |
| SOD-1 | 1:1000 | Santa Cruz Biotechnology | sc-11407 |
| SOD-2 | 1:1000 | Santa Cruz Biotechnology | sc-30080 |
| Catalase | 1:1000 | Santa Cruz Biotechnology | sc-50508 |
| Cytochrome *c* | 1:1000 | Cell signalling | #4272 |
| GRP-78 | 1:1000 | Santa Cruz Biotechnology | sc-13968 |
| IRE1α | 1:1000 | Santa Cruz Biotechnology | sc-20790 |
| PERK | 1:1000 | Santa Cruz Biotechnology | sc-13073 |
| p-eIF-2α | 1:1000 | Santa Cruz Biotechnology | sc-12412 |
| CHOP | 1:1000 | Santa Cruz Biotechnology | sc-793 |
| Calnexin | 1:1000 | Enzo | ADI-SPA-865 |
| JNK | 1:1000 | Santa Cruz Biotechnology | sc-571 |
| p-JNK | 1:1000 | Santa Cruz Biotechnology | sc-12882 |
| p38α | 1:1000 | Santa Cruz Biotechnology | sc-535 |
| p-P38α | 1:1000 | Santa Cruz Biotechnology | sc-17852-R |
| p53 | 1:1000 | Santa Cruz Biotechnology | sc-6243 |
| Bcl-2 | 1:1000 | Cell signalling | #2876 |
| Bax | 1:1000 | Cell signalling | #2772 |
| BID | 1:1000 | Santa Cruz Biotechnology | sc-11423 |
| Casp-9 | 1:2000 | Abcam | ab2014 |
| Casp-3 | 1:1000 | Santa Cruz Biotechnology | sc-7148 |
| PARP-1 | 1:1000 | Santa Cruz Biotechnology | Sc-7150 |
| PAR | 1:1000 | Millipore | AM80 |
| HMGB1 | 1:1000 | Cell Signalling | #3935 |
| Lamin B | 1:1000 | Santa Cruz Biotechnology | sc-6217 |
| p-AMPK | 1:1000 | Santa Cruz Biotechnology | sc-33524 |
| mTOR | 1:1000 | Santa Cruz Biotechnology | sc-8319 |
| p62 | 1:1000 | Cell Signalling | #5114 |
| PI3K | 1:1000 | Santa Cruz Biotechnology | sc- 1637 |
| ATG-5 | 1:1000 | Cell Signalling | #12994 |
| ATG-7 | 1:1000 | Cell Signalling | #8558 |
| BECN-1 | 1:1000 | Cell Signalling | #3495 |
| LC3I/II | 1:500 | Abcam | ab58610 |
| **Secondary antibodies** | | | |
| Goat anti-rabbit IgG-TR  Goat anti-rabbit-HRP | 1:5000  1:25000 | Santa Cruz  Santa Cruz | sc-2780  sc-2004 |

| Gene | Direction | Sequences |
| --- | --- | --- |
| Cyt *c* | Forward | 5’- CAA CAC CTC TCA CAT CTT AC-3’ |
|  | Reverse | 5’-TCC CCA ATC AAA TAC ACA GTT-3’ |
| Casp-3 | Forward | 5’- GTG CTA CAA TGC CCC TGG AT-3’ |
|  | Reverse | 5’- GCC CAT TCA TTT ATT GCT TTC C-3’ |
| PARP-1 | Forward | 5’-TTC ACA TAT CAG CAA GTT ACC-3’ |
|  | Reverse | 5’-CCT GAG CAA TAT CAT AGA CAA T-3’ |
| BECN-1 | Forward | 5’-TGG CAC AAT CAA TAA CTT CA-3’ |
|  | Reverse | 5’-TAA GGA ACA AGT CGG TAT CT-3’ |
| LC3 | Forward | 5’-GAG GTG TAT GAG AGT GAG AA-3’ |
|  | Reverse | 5’-CTG TGA TTG GAT GAA CTG AT-3’ |
| β-actin | Forward | 5’-AAC TAC CTT CAA CTC CAT CA-3’ |
|  | Reverse | 5’-GAG CAA TGA TCT TGA TCT TCA-3’ |

**Table S2.** Sequences of RT-PCR oligonucleotide primers specific for human Cyt *c*, Casp-3, PARP-1, BECN1, LC3 and β-actin.

| Gene | Direction | Sequences |
| --- | --- | --- |
| Atg5 | Sense | 5’- ACGCUAAAAGGCUUACAGUAUCAGA-3’ |
|  | Antisense | 5’-UCUGAUACUGUAAGCCUUUUAGCGUAC-3’ |

**Table S3.** Sequences of siRNA for Atg5.


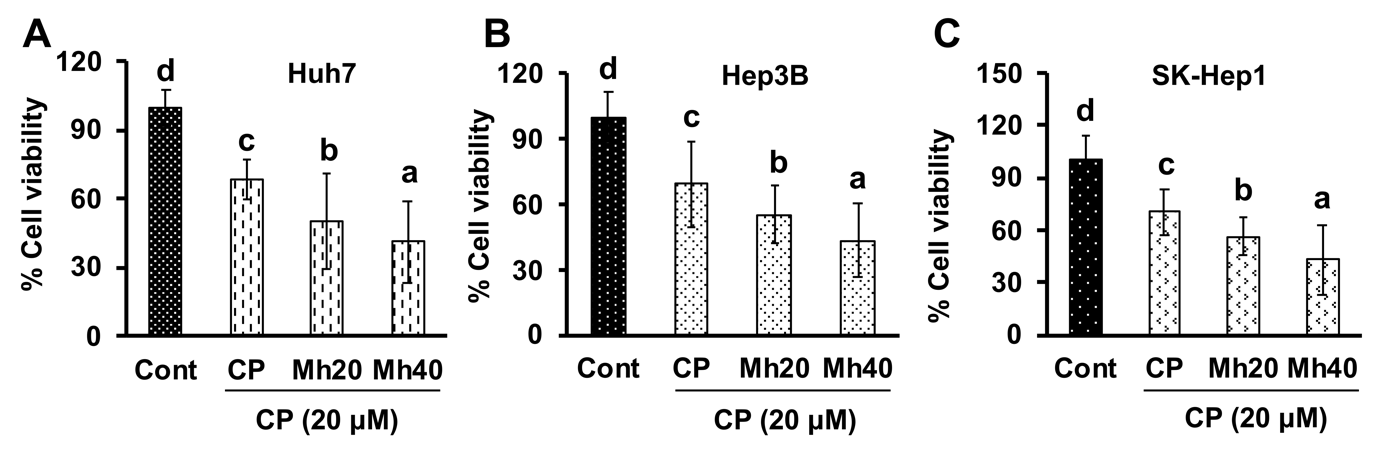


**Figure S1.** Cytotoxic effect of CP and CP-Mh on hepatoma cells. **(A)** Huh7 cells viability measurement after corresponding drug treatment by MTT assay. **(B)** Hep3B cells viability measurement after corresponding drug treatment. **(C)** SK-Hep1 cells viability measurement after corresponding drug treatment. The data are represented as means standard deviation (n = 3). Values of different letters (a–d) differ significantly from each other (p < 0.05).


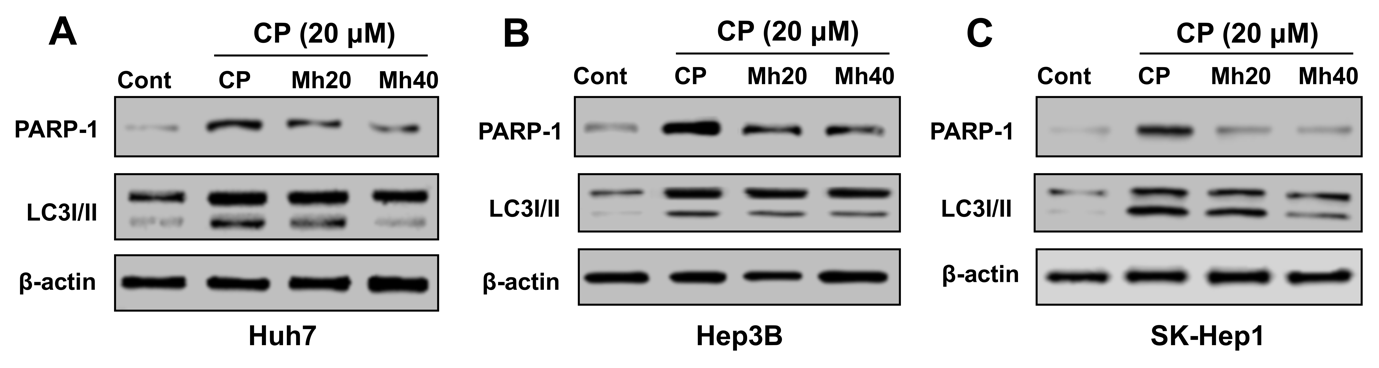


**Figure S2.** Regulatory effect of CP and CP-Mh on PARP-1/LC3I/II protein expression. **(A)** Western blot analysis of PARP-1 and LC3I/II using specific antibodies after corresponding drugs treatment in Huh7 cells. **(B)** The expression of PARP-1 and LC3I/II using specific antibodies after corresponding drugs treatment in Hep3B cells was analyzed. **(C)** The expression of PARP-1 and LC3I/II using specific antibodies after corresponding drugs treatment in SK-Hep1 cells was analyzed. β-actin was used as an internal control.
